# The effectiveness of Technology-assisted Cascade Training and Supervision of community health workers in delivering the Thinking Healthy Program for perinatal depression in a post-conflict area of Pakistan – study protocol for a randomized controlled trial

**DOI:** 10.1186/s13063-016-1308-2

**Published:** 2016-04-06

**Authors:** Shamsa Zafar, Siham Sikander, Syed Usman Hamdani, Najia Atif, Parveen Akhtar, Huma Nazir, Joanna Maselko, Atif Rahman

**Affiliations:** Human Development Research Foundation and Health Services Academy, Islamabad, Pakistan; Human Development Research Foundation, Islamabad, Pakistan; Duke University, Islamabad, Pakistan; Institute of Psychology, Health and Society, University of Liverpool, Manchester, UK

**Keywords:** Thinking Healthy Program, Conflict settings, Community health workers, Technology-assisted Cascade Training and Supervision, Psychosocial intervention, Perinatal depression, Low and middle income countries

## Abstract

**Background:**

Rates of perinatal depression in low and middle income countries are reported to be very high. Perinatal depression not only has profound impact on women’s health, disability and functioning, it is associated with poor child health outcomes such as pre-term birth, under-nutrition and stunting, which ultimately have an adverse trans-generational impact. There is strong evidence in the medical literature that perinatal depression can be effectively managed with psychological treatments delivered by non-specialists. Our previous research in Pakistan led to the development of a successful perinatal depression intervention, the Thinking Healthy Program (THP). The THP is a psychological treatment delivered by community health workers. The burden of perinatal depression can be reduced through scale-up of this proven intervention; however, training of health workers at scale is a major barrier. To enhance access to such interventions there is a need to look at technological solutions to training and supervision.

**Methods/design:**

This is a non-inferiority, single-blinded randomized controlled trial. Eighty community health workers called Lady Health Workers (LHWs) working in a post-conflict rural area in Pakistan (Swat) will be recruited through the LHW program. LHWs will be randomly allocated to Technology-assisted Cascade Training and Supervision (TACTS) or to specialist-delivered training (40 in each group). The TACTS group will receive training in THP through LHW supervisors using a tablet-based training package, whereas the comparison group will receive training directly from mental health specialists. Our hypothesis is that both groups will achieve equal competence. Primary outcome measure will be competence of health workers at delivering THP using a modified ENhancing Assessment of Common Therapeutic factors (ENACT) rating scale immediately post training and after 3 months of supervision. Independent assessors will be blinded to the LHW allocation status.

**Discussion:**

Women living in post-conflict areas are at higher risk of depression compared to the general population. Implementation of evidence-based interventions for depression in such situations is a challenge because health systems are weak and human resources are scarce. The key innovation to be tested in this trial is a Technology-assisted Cascade Training and Supervision system to assist scale-up of the THP.

**Trial registration:**

Registered with ClinicalTrials.gov as GCC-THP-TACTS-2015, Identifier: NCT02644902.

## Background

The prevalence of maternal depression in low and middle income countries (LMICs) ranges between 18 and 25 % [1]. In Pakistan the mean prevalence of depression is 33 %, with women being at a greater risk than men [2]. Untreated depression in women is of particular concern due to its adverse effects on the health of the mother and infant. Maternal depression, is linked with pre-term birth [3], low birth weight [3], under-nutrition in the first year of life [4], higher rates of diarrhea [5], and early cessation of breastfeeding [6]. Maternal depression has a profound adverse trans-generational impact [6]. The economic burden of depression in pregnant and postnatal women not only includes the cost of treating depression, but the cost of complications, such as pre-term birth and low birth weight [3]. Children of depressed mothers also have higher lifetime medical spending due to the adverse effects of postpartum depression on the child’s own health [7]. The scope and magnitude of the problem is magnified in humanitarian crisis settings [8].

Evidence-based interventions for depression exist [9] but there is slow progress in the “know-do gap”: the gap between what is known and what gets implemented in LMICs [10, 11]. The burden of illness can be reduced by narrowing this gap through scale-up of proven interventions. The Thinking Healthy Program (THP) is a culturally adapted [12] cognitive behaviour therapy (CBT)-based psychosocial intervention for maternal depression, which relies on CHWs called Lady Health Workers (LHWs) for its delivery [12, 13]. In a recent meta-analysis [9], THP was shown to have one of the largest effect sizes and has been adopted by the World Health Organization (WHO) for global dissemination through its mental health Gap Action Program (mhGAP) [14].

The major issues in scaling up the coverage of health interventions include the costs, equity and quality concerns and service delivery issues [15]. In the context of scaling up, the supply of additional human resources is a major barrier [16]. Another challenge in the scaling up of this evidence-based intervention is the provision of training and supervision at scale, especially in post-conflict areas with weak health systems. We aim to meet this challenge by providing technology-based solutions to training and supervision. Building on our previous work in this area, we aim to develop a Technology-assisted Cascade Training and Supervision for the THP (TACTS-THP) system that includes a tablet-based multimedia manual, using “Avatar” characters, allowing standardized training to be delivered without the need for a specialist trainer; and a cascade training model whereby specialists supervise the LHW program supervisors from a distance, who in turn supervise the LHWs as part of their routine. The cascade training has been found to be effective in training non-specialists to deliver mental health interventions [17].

The LHW program covers 85 % of Pakistan’s rural population by utilizing 115,000 LHWs. If we are able to provide a technological solution to their training and supervision, LHWs have the potential to provide treatment to one in four women with perinatal depression in rural Pakistan. The THP-TACTS system will be widely disseminated. If proven effective the technology has the potential to be replicated for scale-up of other mental health interventions.

### Primary objective

The primary objective of this trial is to demonstrate whether LHWs in Pakistan attain the same competence level for delivering THP to depressed mothers if they are trained with the help of technology versus training by specialists.

### Secondary objective

The secondary objectives of this trial are the assessment of feasibility and acceptability of the technological training and supervision approach by LHWs and community.

### Hypothesis

LHWs trained through TACTS will be as competent in the skills needed to deliver THP as those trained by specialists.

## Methods/design

### Design

The study is a non-inferiority, single-blinded randomized controlled trial with two arms, comparing the effectiveness of two training strategies for the THP. Consolidated Standards of Reporting Trials (CONSORT) guidelines [18] will be followed in the design, implementation and reporting of this randomized controlled trial.

### Settings

We intend conducting the study in the Swat district in Northern Pakistan which has been affected by multiple humanitarian crises in recent years. Swat has a population of 1,257,602 persons, 86 % of whom live in rural areas with the average household size of 8.8 persons with a low female literacy. The resurgence of the Taliban movement in the Swat Valley of Pakistan since 2004, and the military operations in 2009 and the displacement of residents has created a humanitarian crisis in the area [19]. The devastating floods of 2010 led to thousands of people losing of their homes, and caused destruction to houses, roads, schools and health facilities [20, 21]. A recent community-based cross-sectional survey of 349 pregnant women in Swat showed that the prevalence of current psychological distress was 38.1 % [22]. Mental health impacts of conflict are often severe [8, 23] and may not be depicted in the national statistics. The health infrastructure of a post-conflict zone like Swat is particularly fragile with the health workers under threat of being attacked, thus compromising their training and supervision. The district has a functioning LHW program and LHWs, who have 10–14 years of education and provide preventive primary care services in the community, have the capacity to use technology. A Lady Health Supervisor (LHS) supervises 15–20 LHWs in one supervisory zone.

### Participant recruitment

The LHW program will be approached in the Swat district of Pakistan to obtain a list of the LHSs and LHWs who are currently working in the six LHS supervisory zones. Since Swat has extreme winter weather making some parts inaccessible in winters, the supervisory zones will be selected on the basis of accessibility throughout the year. The LHW program will be asked to inform all the LHSs and LHWs in the zones about the study. By simple random sampling 80 LHWs will be selected for the study and written informed consent will be obtained by the research team from the LHWs.

### Sample size estimation

The primary outcome of the study is the mean competence scores post training and at 3 months. We define non-inferiority as a difference of 5 points or less (corresponding to a 10 % difference on the outcome measure score) in the mean competence score between the two groups. A sample size of 80 LHWs (40 LHWs per arm) will have 99 % power to detect a 5-point margin with a 0.05 one-sided alpha level. Even after potential attrition in this area, at 3 months we will need 30 health workers in each group for a power of 80 % and a two-sided alpha error of 0.05.

### Randomization

**Fig. 1:**
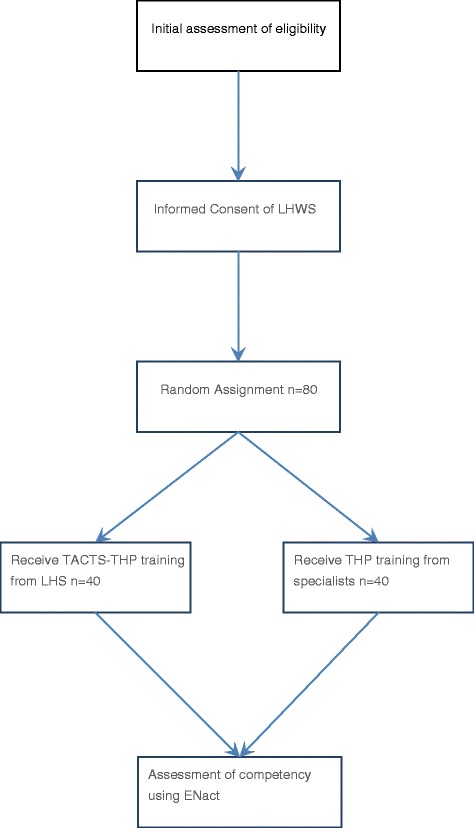
Randomization for Think Healthy Program-Technology-assisted Cascade Training and Supervision (THP-TACTS)

The selected LHWs will be randomly allocated to the technology experimental arm or the specialist group control arm. Randomization will be conducted by an individual not involved in the study by using computer-generated random numbers (Fig. 1).

### The intervention – TACTS

A tablet-based multimedia manual is developed for THP training and supervision purposes. The intervention material is divided into five training sessions, which are converted into narrative scripts by a panel of THP experts. The narratives have incorporated all the core techniques and principles of the intervention such as effective use of counseling skills, collaboration with the mothers’ families, guided discovery (style of questioning to probe mother’s health beliefs), and setting health-related tasks. Culturally appropriate real-life characters for the trainers and the trainees have been developed. An artist has converted the characters into “Avatars” (graphic image representing each character) (Fig. 1), which were used to voice the narrative scripts. Our group had successfully used this methodology of Technology-assisted Cascade Training (TACT) for training parents of children with developmental disorders [22]. The TACTS has also used case scenarios and role plays to reinforce key messages and training skills. For example, the role plays have demonstrated: sessions’ delivery by THP-trained LHWs to depressed mothers, dealing with challenging situations and dealing with adverse events. The training has been made interactive through suggesting activities for the actual trainees using a pause button and instructions. These activities are aimed to help them practice delivery of sessions through the role plays, peer assessment of role plays, reflecting on one’s learning, sharing relevant personal and work experiences, and practicing problem-solving strategies. A separate module on supervision has been developed for the LHSs to guide them on conducting supervisions. The software has the provision to modify and update the training scenarios. The software will be tested with LHSs and academics during and after the development process. The qualitative feedback regarding multiple aspects of the system and the user interface will be recorded.

**Fig. 2:**
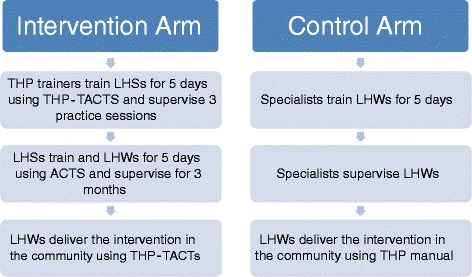
Training for the Think Healthy Program (THP): intervention versus control arm

In the intervention arm THP trainers (who have experience of delivering THP for 1 year) will give 5 days’ training to the LHS on THP-TACTS and will supervise three practice cases delivered by the LHSs. The LHSs will then cascade this training using THP-TACTS to train LHWs in 5-day training sessions (Fig 2).

### Control – specialist-delivered training

The control group of LHWs will be directly trained by specialists in a 5-day training program and supervised as in the original THP. Specialists are experienced mental health academics who have a clinical background of delivering CBT and THP. The LHWs in both arms will deliver the intervention in the community to depressed women using the original THP manuals. Five LHWs will deliver the TACTS intervention using a tablet.

### Potential biases

#### Observer blinding

The raters who will assess the competence level of LHWs post training and at 3 months will not be aware of the allocation status of the training methods of the LHWs.

#### Contamination

The THP group supervision for LHWs will be held monthly, in addition to their routine monthly meeting. The LHWs will be advised not to disclose information to their colleagues.

#### Primary outcome – competence of health workers

Primary outcome will be measure of LHW competence in the two groups. Equivalence is defined as a difference of less than 5 points (10 %) in the mean score of the competence measure across the two groups.

##### Outcome measure

The primary outcome of competence will be measured by using the ENhancing Assessment of Common Therapeutic factors (ENACT) scoring system [24]. ENACT is an 18-item scoring system for training and supervision across settings in mental health for global use. ENACT was developed by a process of selecting items through item generation, item relevance, and item utility and piloted in trainings for psychological treatments and the mhGAP. Inter-rater reliability was acceptable for non-specialists [24].

The key areas assessed in this tool are: verbal and non-verbal communication; rapport building; empathy; family involvement; goal setting; exploration of feelings; social support; functioning; life events; coping; mental health problems; promoting hope; problem solving; feedback; confidentiality and assessment of harm. The items are scored from 1 to 3, where 1 = needs improvement, 2 = partially done, 3 = done well and the final score ranges from 18 to 54. A score of at least 2 is required for all domains to achieve competence. A score of 43 represents a satisfactory level of competence and corresponds to 80 % of the total score. This tool can be used for role plays as described in the paper [24].

We will use ENACT in both control and intervention arms immediately after training and at 3 months. Independent outcome assessors, who are blinded to the training methodology used, will assess the LHWs on standardized patient role plays. These assessors will be trained by the THP trainers using the ENACT instrument, using role plays. Raters who performed the post-evaluation assessments will rate live observations of LHWs in the household settings using ENACT checklists after 3 months of training. We expect competency scores to increase as the LHWs gain more practice implementing the THP.

#### Secondary outcomes

##### Cost evaluation

We will take the public health sector and program perspective in the cost evaluation of the program. The objective of the evaluation will be to find out if training of LHWs in the evidence-based program using technology (THP through TACTS) is more cost-effective compared with the training of LHWs by the specialists using the conventional model (specialists training LHWs in the THP). To demonstrate this we will collect the data on (1) direct costs associated with training of LHWs in THP using the TACTS system, and (2) Information on the costs associated with the training and support of LHWs in the THP by the specialists following the conventional model. We will also collect data on the opportunity costs associated with the specialists’ time. The information will be gathered through focused discussions held between the trainers and the research team and data will be collected on the way the training was delivered, including the venue of the training (the training space used), and the average number of hours worked by the specialists, LHSs, THP trainers, and LHWs. Data collection will continue throughout the study period. Information will also be collected on the cost of developing TACTS and other related costs, e.g., communication costs, logistics costs, training material and stationary.

Although cost data are often skewed, analyses will compare the mean costs in the two groups using a standard *t* test with ordinary least squares regression used for adjusted analyses and the validity of results confirmed using bootstrapping [25]. Subgroup analyses by baseline ENACT score will be performed using tests of interaction.

##### Qualitative evaluation – intervention feasibility

The qualitative evaluation will include exploring the views of health workers on training and supervision, acceptability of the content, and any challenges in implementation 3 months post training. Focus group discussions (FGDs) (*n* = 4) with LHWs and one FGD with LHSs will be conducted. Independent assessors having experience with qualitative data collection will conduct FGDs. They will be audio recorded and field notes will be taken. Data on LHW demographic variables, including age, experience, education, etc., will be collected.

#### Statistical analysis

The competence scores of the two groups will be compared using Student’s *t* test post training and at 3 months. A difference of less than 5 points between the two groups will be interpreted as supporting the non-inferiority hypothesis. We will also examine whether the competence scores improve at the 3-month mark after supervision. Stata software will be used for this. Qualitative data will be analyzed using framework analysis and manual software for qualitative studies. Cost-effectiveness will be assessed by combining costs with the primary outcome measure in incremental cost-effectiveness analysis. In addition, repeat re-sampling from the costs and effectiveness data (bootstrapping) will be used to calculate the probability that each of the training methods is the optimal choice, subject to a range of possible maximum values (ceiling ratio) that a decision-maker might be willing to pay for a unit improvement in ENACT score. A cost-effectiveness acceptability curve will be presented by plotting these probabilities for a range of possible values of the ceiling ratio [26].

#### Data management

Data management will be done by the Human Development Research Foundation (HDRF). All participating raters will hand over their collected information to the field coordinator. Data will be checked by the field coordinator on site for plausibility, consistency, and completeness. Any missing data or inconsistencies will be reported back to the assessor and clarified. Unique identification (ID) numbers will be used and individual LHWs will not be identifiable in the dataset. Data will then be exported and stored, and backed up on a secure server at the HDRF.

#### Ethical considerations

The study protocol was approved by the Ethics Committee of the Human Development Research Foundation, Pakistan (IRB/001/2015). The Ethics Committee is registered with the Office for Human Research Protection (OHRP), Rockville, MD, USA. The committee stated that there are no legal or ethical concerns to the planned procedures. The trial was registered in the ClinicalTrials.gov, 2016 (Identifier: NCT02644902).

#### Trial management

The trial management will be overseen by two committees: (1) the Trial Management Committee (TMC), which will monitor all aspects of conduct and implementation of the trial and its progress. The TMC will meet weekly initially, especially during the startup phase of the trial, and every second week thereafter if they decide it to be necessary. Minutes of TMC meetings will be kept; (2) the Trial Steering Committee (TSC) will chart the overall progress of the trial and will also provide technical input on the overall trial protocol. The TSC will also oversee trial participant’s safety and data monitoring. The TSC will meet every 6 months and minutes of key decisions will be maintained. The TMC is composed of the principal investigator (PI), co-investigators, data manager/trial manager, and the site team (research associate and field coordinators). The TSC is composed of the PIs, study co-investigators, the trial manager, and the study statistician. The TSC must agree on the final trial protocol and any protocol amendments and will provide advice on all aspects of the project. Decisions about any substantial amendments to be made to the protocol will be the responsibility of the TSC.

#### Ethical considerations

The study has been granted ethical approval from the Ethics Committee of the HDRF. Any updates/amendments made to the trial protocol will be shared with the Ethics Committee. For enrollment informed consent is mandatory (written or witnessed if the participant is illiterate). The confidentiality of personal data will be protected through data anonymity and through procedures to separate study data and participant identifiable data. If a woman is found to be seriously depressed by the LHWs, she will be referred to a psychiatrist and this will be reported to the Ethics Committee of the HDRF.

## Discussion

The development of THP-TACTS is an innovative and comprehensive approach to improving the scale-up and implementation of THP in the community. Developing such technology-assisted tools creates an opportunity to train CHWs at scale and to substantially increase the number of women with depression who do not have access to specialists in mental health. A demonstration of the effectiveness of TACTS in attaining health worker competence through this research study may result in greater availability of this technology not only in other humanitarian settings but also where access to mental health services is an issue. TACTS, if cost-effective in the long term, will help to strengthen the capacity of primary care systems to address multiple health priorities using technology. To our knowledge, this is the first effectiveness study on the technology-based training of CBT and THP for perinatal depression by a non-specialist. Additionally, as a feasibility study we will document any barriers to acceptability of this training program, enabling us to develop guidelines that will maximize successful implementation of the program in the future.

## Trial status

Enrollment for the trial has not yet started.

## Abbreviations

TACTS: Technology-assisted Cascade Training and Supervision
CHWs: community health workers
ENACT: ENhancing Assessment of Common Therapeutic factors
HDRF: Human Development Research Foundation
LHS: Lady Health Supervisor
LHW: Lady Health Worker
LMICs: low and middle income countries
mnGAP: mental health Gap Action Program
THP: Thinking Healthy Program
